# Assessing the impact of humidex on HFMD in Guangdong Province and its variability across social-economic status and age groups

**DOI:** 10.1038/srep18965

**Published:** 2016-01-08

**Authors:** Wangjian Zhang, Zhicheng Du, Dingmei Zhang, Shicheng Yu, Yong Huang, Yuantao Hao

**Affiliations:** 1 Department of Medical Statistics and Epidemiology & Health Information Research Center & Guangdong Key Laboratory of Medicine, School of Public Health, Sun Yat-sen University, Guangzhou 510080, Guangdong Province, China; 2 Chinese Center for Disease Control and Prevention, Beijing 102206, China; 3 Guangzhou Center for Disease Control and Prevention, Guangzhou 510440, China

## Abstract

Humidex is a meteorological index that combines the impacts of temperature and humidity, and is directly comparable with dry temperature in degrees Celsius. However, to date, no research has focused on the effect of humidex on hand, foot and mouth disease (HFMD). The current study was designed to address this research need. Case-based HFMD surveillance data and daily meteorological data collected between 2010 and 2012 was obtained from the China CDC and the National Meteorological Information Center, respectively. Distributed lag nonlinear models were applied to assess the impact of humidex on HFMD among children under 15 years oldin Guangdong, and its variability across social-economic status and age groups. We found that relative risk (*RR)* largely increased with humidex. Lag-specific and cumulative humidex-*RR* curves for children from the Pearl-River Delta Region as well as older children were more likely to show two-peak distribution patterns. One *RR* peak occurred at a humidex of between 15 and 20, and the other occurred between 30 and 35. This study provides a comprehensive picture of the impact of humidex on HFMD incidence in Guangdong Province. Results from the present study should be important in the development of area-and-age-targeted control programs.

Hand, foot and mouth disease (HFMD) is a common childhood infection and has become a major public health issue in China, affecting over two million persons annually^1^,^2^. It is a particular concern in Guangdong Province, where incidence is higher than four times the national average and exceeded 30 cases per 10, 000 persons in 2012^3^,^4^. Considerable research has focused on the role of meteorological factors such as temperature, sunshine and relative humidity in HFMD development^5^,^6^,^7^,^8^.

Impacts of temperature and relative humidity on HFMD are the most-commonly reported foci in the literature. Most of these studies assessed the impact of one predictor after controlling for the effect of the other. For instance, in a previous study, a positive association between relative risk of HFMD and temperature was observed until 25 °C, with relative humidity being controlled as a confounding factor^9^. However, results from these studies should be interpreted with caution. As stated by Canadian meteorologists, the same temperature can give people completely different feelings under different humidity conditions^10^,^11^. An index that combines the effects of temperature and humidity is therefore preferred. Humidex is such a comprehensive index^11^,^12^,^13^,^14^.Nowadays, variations of humidex are used around the world^15^. However, to date, no research has examined the effect of humidex on HFMD. The present study was designed to help address this research gap.

Meteorological factors tend to have nonlinear impacts on HFMD^16^. That is, the rate of change of an outcome tends to change with meteorological predictors (i.e., the rate of change of the relative risk (*RR)* may change with humidex in the present study). In assessing predictor-HFMD relationships, there have been numerous attempts to quantify delayed impacts, since meteorological factors (i.e. humidex in this study) may affect the occurrence of HFMD for a period lasting some days after their occurrence^17^,^18^. Time series analyses such as distributed lag nonlinear models are particularly suitable in such cases^19^.

The assessment of the impacts of meteorological factors on HFMD is further complicated by several additional factors. First, socio-economic status is considered a potential confounder. Developed areas are more densely populated and tend to face heavier traffic and more social contact, consequently bearing higher HFMD burdens. As reported, the developed Pearl-River Delta Region has a higher burden of HFMD relative to the developing Non-Pearl-River Delta Region within Guangdong province^3^. Impacts of meteorological factors (i.e. humidex in this study) on HFMD may vary across different regions, although no comparative studies have addressed this issue. Second, age is another commonly studied confounder. Children of different age groups tend to be differentially susceptible and to have different life styles and social contacts. Therefore, their responses to meteorological conditions may be significantly different^20^,^21^. When stratified by potential confounders, results may vary substantially.

This study aimed to quantify the impact of humidex on childhood HFMD in Guangdong Province, China, from 2010 to 2012, and to assess its variability across social-economic status and age groups.

## Results

### Descriptive statistics

**Figure 1:**
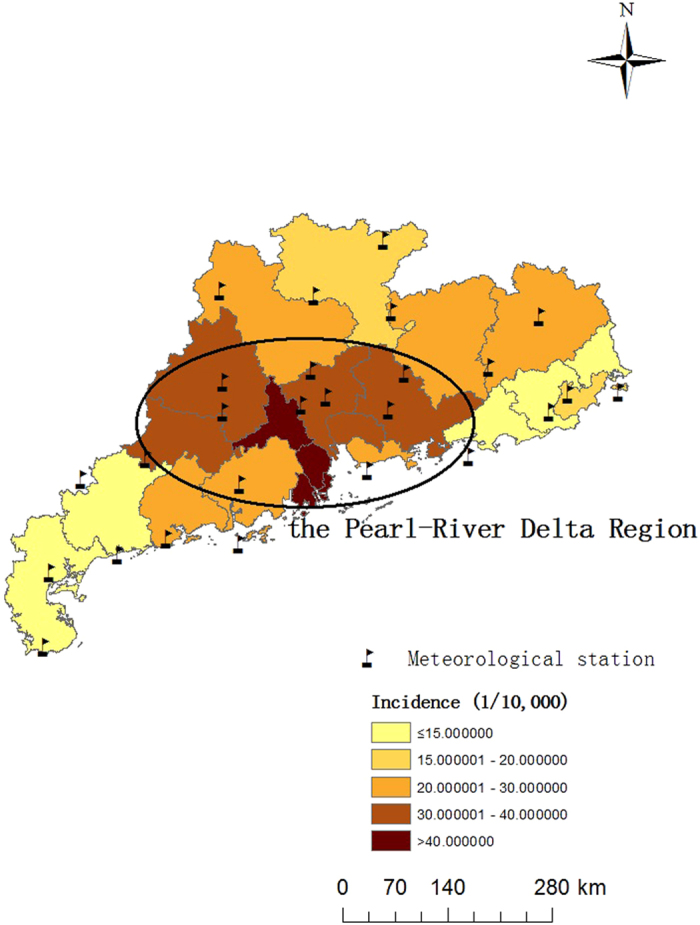
Spatial distribution of meteorological stations and the average incidence of HFMD in Guangdong during 2010–2012 (The map was created with ArcGIS10.0, ESRI).

There were in total 827,911 cases of HFMD under 15 years old reported to the case-based surveillance system (CBSS) during the period of 2010–2012 in Guangdong province (Table 1).The average provincial incidence during the study period was 26.26 cases per 10,000 persons. Incidence in the Pearl-River Delta Region (34.17 cases per 10,000 persons) was greater than twice that in the Non-Pearl-River Delta Region (17.07 cases per 10,000 persons) (Fig. 1).

**Figure 2:**
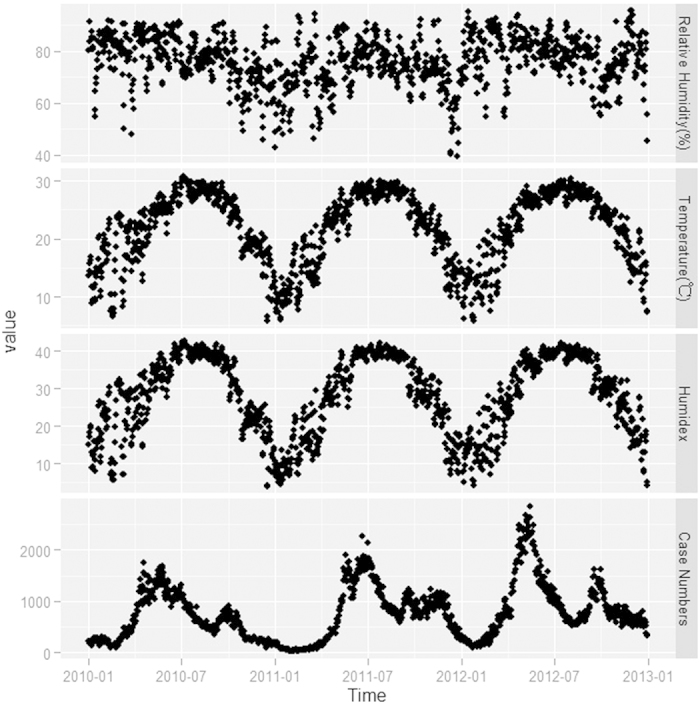
Time series plot for reported HFMD cases and three main meteorological variables in Guangdong from 2010 to 2012.

The provincial relative humidity and temperature were 76.30% and 21.75 °C on average respectively, which demonstrates typically subtropical climate characteristics (Table 1). Humidex in the current study had a seasonal trend similar to the time series of temperature (Fig. 2). However, humidex range was much wider than that for temperature. The peak temperature was around 30 °C, whereas the peak humidex was above 40. Because of the high humidity in Guangdong, the human-perceived equivalent temperature was much higher than dry temperatures reported.

Time series of the case number and humidex showed similar seasonality (Fig. 2).

### Predictor-response relationship

**Figure 3:**
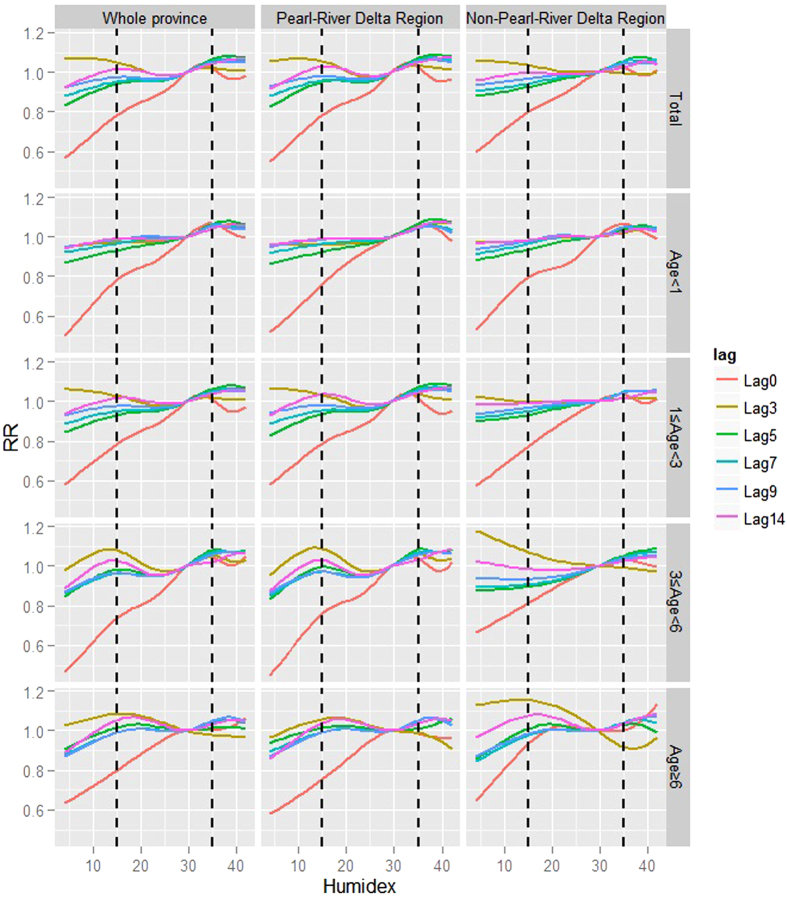
Lag-specific humidex-*RR* curves at various lags.

In examining lag-specific humidex-HFMD relationships, *RR*s mainly increased with humidex (Top-left panel, Fig. 3). Two peaks could be found on most of the humidex-*RR* curves except the curve for the current day (i.e., 0 days of lag), which had a relatively greater slope and kept going upward until 33. One peak occurred at a humidex between 15 and 20, whereas the other was between 30 and 35. After the second peak, curves tended to become flat or decline slightly.

In assessing the cumulative impact of humidex on HFMD over a 14-day period, a similar pattern was observed, with *RR* increasing and peaking successively at 16 and 36. After the second peak, the humidex-*RR* curve was going slightly downward.

**Figure 4:**
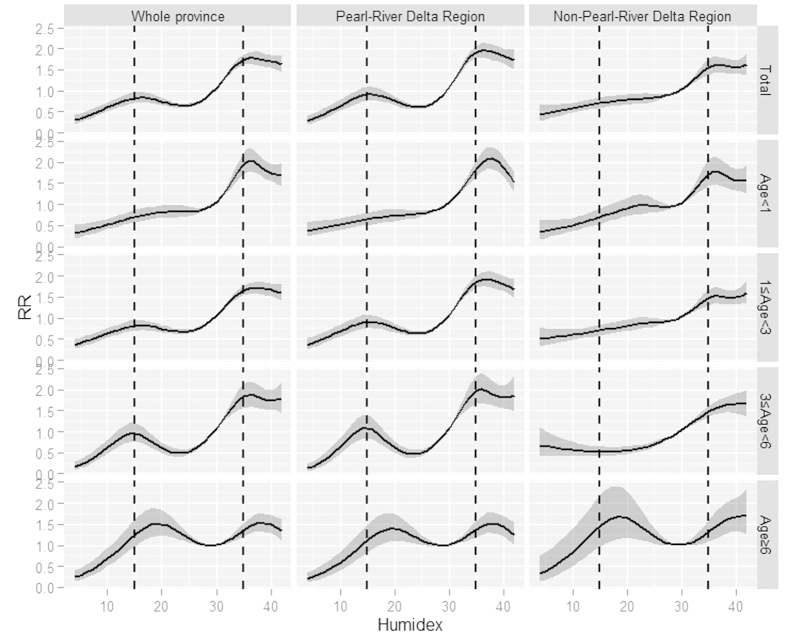
Cumulative impacts of humidex on HFMD over a 14-day period.

We also considered variability in the humidex-HFMD relationship across different regions and age groups. The humidex-*RR* curve for the Pearl-River Delta Region tended to have two peaks. One was at 15 and the other was at 36. The Non-Pearl-River Delta Region only had a single *RR* peak at 36 (first row of the panel, Fig. 4).

When comparing curves for different age groups, *RR*s for children under 1 year old only peaked at around 36, whereas *RR*s for children greater than 1 year old tended to show two-peak distribution patterns, with this more apparent for older age groups (first column of the panel, Fig. 4).

Socio-economic indices might show interaction effects with age groups on humidex impact. For the Pearl-River Delta Region, humidex-HFMD relationships were very similar to the provincial situations. But for the Non-Pearl-River Delta Region, only children greater than 6 years old had two *RR* peaks, successively at humidex scores of 18 and 40.

### Lag effect assessment

**Figure 5:**
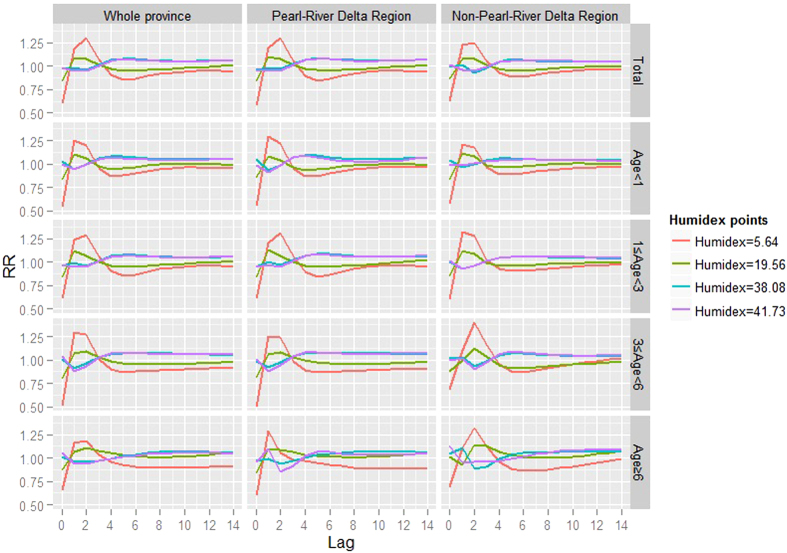
The lag structure of humidex-HFMD relationships.

In the present study, where a humidex of 29.51 was selected as the reference, peak vulnerability to low humidex (e.g., the 1^st^ or 25^th^ percentile of the provincial humidex distribution during the study period) occurred 1 or 2 days afterward (Fig. 5). After the peak, *RR*s declined to relatively low levels, which was termed the “harvest effect” or “displacement”, suggesting that a portion of those who caught HFMD would had been ill soon thereafter. Lag effects of high humidex (e.g. the 75^th^ or 99^th^ percentile of the provincial humidex distribution during the study period) were in an opposite direction to that of the overall relationship, with troughs occurring at 1 or 2 days of lag (Fig. 5).

Tendencies of lag-*RR* curves of different subsets were similar except for peak or valley timing, which varied slightly across regions and age groups.

## Discussion

In recent years, HFMD has been increasingly recognized as a public health priority, particularly for children under 15 years old^9^,^16^. As reported, in China there were around 2.78 million cases of HFMD reported to the CBSS during the single year of 2014^1^. Because of the absence of targeted vaccination or specific treatments, identifying environmental predictors which have significant impacts on HFMD is of great importance^22^. The present research builds upon prior studies by modeling the relationship between HFMD and the never before studied in this context humidex, a meteorological index that comprehensively reflects the impacts of humidity and temperature, with distributed lag non-linear models. In addition, this was also the first research conducted in Southern China to assess the variability in impact of humidex across socio-economic status and age groups. This study could provide guidance to policy makers, health agencies and local communities in choosing targeted prevention and control strategies, and allocating health resources.

Several key themes emerged from the results of this research. In terms of putative predictors, we confirmed that humidex (the human-perceived equivalent temperature—how hot it feels) was actually higher than dry temperature as recorded by meteorological stations. The human body normally cools itself by perspiration, or sweating. However, relative humidity would work to reduce this heat-removal procedure, resulting in a sensation of being overheated^10^,^11^. This situation is particularly apparent in humid subtropical areas like Guangdong. Therefore, information derived from this research could ultimately be more useful than previous temperature-based conclusions.

In terms of overall vulnerabilities, a significant positive correlation was observed between HFMD and humidex. This result supports most previous research, showing that the relative risk of HFMD largely increases with temperature-related indices^6^,^23^,^24^,^25^. However, most of these studies were based on the assumption that predictors have linear impacts on HFMD incidence, and quantified average change in outcomes that are caused by one-unit increases in predictors. Recently, the introduction of smoothing functions and the development of semiparametric models like generalized addictive models (GAM) and distributed lag nonlinear models (DLNM) have made the assessment of nonlinear impacts of meteorological factors on HFMD more feasible^9^,^16^,^26^.

With distributed lag nonlinear models, we found that there exist two *RR* peaks for the cumulative impact curve and for most of the lag-specific curves, one of which occurred at a humidex of between15 and 20 and the other at around 35. Causes for these two peaks might be different.

One explanation for the first peak might be that perceived temperatures between 15 and 20 are more comfortable relative to other ranges^27^. Therefore, children tended to engage in more outdoor activities, have more social contacts, and consequently, were more likely to be infected with HFMD during these agreeable days.

Regarding the second threshold, a previous study in the same area found that the relationship between HFMD incidence and temperature was characterized by increasing below and flattening above 25 °C^9^. Given the fact that humidex as an index tends to be greater than temperature (as shown above, the maximum temperature was around 30 °C, whereas the peak humidex was above 40), the second threshold detected in the present research should be similar to that identified in the previous study. This threshold might result from interactions among biological mechanisms and human contact behaviors^9^,^28^. For instance, after the first minor peak, the promoting effects of humidex on the proliferation and transmission of HFMD-related enteroviruses might be predominant. Immunity of children might be negatively influenced simultaneously during this process. Some of the humidex-*RR* curves headed downward at the second peak, which might be due to a reduction in outdoor activities because of the great discomfort caused by high humidex^29^.

In terms of interregional and inter-age distinctions, children from the Pearl-River Delta Region and of older age were more likely to experience a minor *RR* peak at humidex between15 and 20. Results from stratified analyses reinforce our speculation on the reason for the first peak,when analyzed as a whole.The Pearl-River Delta Region is much more prosperous, with better public transport and infrastructure^30^. Therefore, children in this area might enjoy more outdoor activities with their families or friends during pleasant days, except for those under 1 year old,who are more likely to spend most of their time indoors. The Non-Pearl-River Delta Region is less developed. Only children greater than 6 years old had elevated risk in this humidex range.

We also found that peak vulnerability to low humidex occurred 1 or 2 days after the weather events, followed by a “harvest effect” or “displacement”. Lag effects of high humidex were in the opposing direction, with troughs occurring at 1 or 2 days lag. However, these results should be interpreted with caution since both the absolute value and relative size of *RR*s at different lags vary with the reference point used (reference = 29.51 in the present research).

This study has several strengths, including the fact that this is, to our knowledge, the first research examining the impact of humidex, an index comprehensively reflecting the effects of humidity and temperature, on HFMD, and to further assess variability of effects across social-economic status and age groups. Distributed lag nonlinear models make it possible to study predictor-HFMD relationships simultaneously at various lags, without co-linearity problems. Nevertheless, several limitations of this study should be acknowledged. First, the study period was limited in 3 years, which is shorter than that of previous studies. To ensure a large enough sample size, analyses were conducted on a daily basis in this study, which was also considered to provide more accurate information on the humidex-HFMD relationships. Second, this study was ecological in nature and bias due to exposure and/or outcome assessment being inevitable rather than manipulated variables. Future research should be carried out to confirm our findings, particularly our speculation on enterovirus survival issues and individual activity changes.

In conclusion, this study provided a comprehensive picture of the impact of humidex on HFMD incidence in the largest province of Southern China. *RR*s mainly increased with humidex, with two peaks observed on most lag-specific humidex-*RR* curves as well as on the cumulative curve. Curves for the Pearl-River Delta Region and older age groups more likely to show two-peak distribution patterns. Results from the present study are practical and potentially important for developing area-and-age -targeted control programs.

## Materials and Methods

### Ethics statement

This study was based on official hand, foot and mouth disease (HFMD) surveillance data in Guangdong. Analyses were conducted at the aggregate level and no confidential information was involved. The research study protocol was approved by the Institutional Review Board of the School of Public Health, Sun Yat-sen University. In the present study, methods were carried out in accordance with the approved guidelines.

### Study site

Guangdong Province, one of the biggest provinces in Southern China, has a population of 104 million (from 2010 census data). According to the characteristics of the natural landscape and social-economic development, Guangdong Province can be generally divided into two parts: the Pearl River Delta Region (areas within the ellipse, as shown in Fig. 1) and the Non-Pearl River Delta Region. According to statistical yearbooks for Guangdong, the Pearl River Delta Region has a higher level of social-economic development, accounting for 80% of GDP in Guangdong Province with less than 50% of the population^30^. The HFMD burden in the Pearl River Delta Region was much higher than that in the other areas (Fig. 1).

### Surveillance data of HFMD

Case-based HFMD surveillance data collected between 2010 and 2012 was obtained from the National Center for Public Health Surveillance and Information Services, China Center for Disease Control and Prevention (China CDC). Information including sex, birth time, onset time and the current address (with ZIP code) was reported to the case-based surveillance system (CBSS) when a patient was diagnosed with HFMD, according to the National Clinic Guide (Version 2009)^31^.

According to our data, over 99.5% HFMD cases were children under 15 years old. Therefore, we focused on the incidence of HFMD among children under 15 years old in this study. Form of child care affects HFMD infection. In China, children under 3 years old are usually cared for at home, 3–6 year olds attend kindergartens, and those above 6 years old go to school. Children under 1 year old obviously differ from those aged 1–3 years in terms of daily activities. To assess the variability of impacts of humidex across age groups, analyses were stratified accordingly (<1, 1–3, 3–6, and >6 years).

Population data was obtained from Statistical Yearbooks of Guangdong.

### Meteorological data

Daily meteorological data was obtained from the National Meteorological Information Center (http://cdc.nmic.cn/). Data from meteorological stations of the same region (i.e. Pearl-River Delta/Non-Pearl-River Delta in stratified analyses and the whole province when analyzed as a whole) was then averaged. Four daily meteorological variables were included in the current study: Relative humidity, temperature, rainfall and wind speed.

Humidex was calculated from relative humidity and temperature with a calculator provided by CSGNetwork (http://www.csgnetwork.com/) in California. Humidex, developed by Environment Canada, is an index that combines the effect of temperature and humidity. It is unitless but equivalent to dry temperature in degrees Celsius. For example, if the temperature is 30 °C, and the calculated humidex is 40, then it indicates that the humid heat feels approximately like a dry temperature of 40 °C^14^.

### Statistical analysis

Distributed lag nonlinear models with quasi-Poisson distribution were applied to quantify the effect of humidex on childhood HFMD, with daily counts of HFMD cases as the dependent variable and humidex as the predictor. The model used in this research was


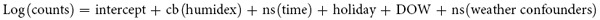


Counts was the daily HFMD cases, assuming a quasi-Poisson distribution; cb(humidex) was the cross-basis for the space of the predictor and the lag dimension of daily humidex. The median value for provincial daily humidex was used as the reference value for *RR* calculations. ns(time) was a natural spline fit to the days of the year; holiday and DOW were dummy variables representing whether a given day was a holiday and day of week, respectively; ns(weather confounders) was short for three natural spline fits to sunshine, rainfall and wind speed, all with 3 degrees of freedom (df). Relative humidity and temperature were excluded to avoid co-linearity problems.

Based on previous studies, we specified the lagged effect of humidex up to two weeks of lag (minimum lag equal to 0 by default)^9^,^22^,^32^, and studied the lag-specific humidex-*RR* relationships and the cumulative humidex-*RR* relationship, simultaneously. Df for ns(time) was specified within 5–10, and for both the space of the predictor and the lag dimension of daily humidex within 3–6. Sensitivity analyses were conducted to evaluate the robustness of assessments and select the “best parameters” according to Akaike’s Information Criterion for quasi-Poisson (Q-AIC)^19^,^33^,^34^.

Data manipulation and analyses were conducted with R packages including “data.table”, “ggplot2” and “dlnm”.

## Additional Information

**How to cite this article**: Zhang, W. *et al.* Assessing the impact of humidex on HFMD in Guangdong Province and its variability across social-economic status and age groups. *Sci. Rep.* **6**, 18965; doi: 10.1038/srep18965 (2016).

## Figures and Tables

**Table 1:** Descriptions of HFMD cases and three main meteorological variables.

	HFMD Cases(N)	Meteorological factors						
		Relative Humidity (%)	Temperature (°C)	Humidex				
				1%	25%	50%	75%	99%
The whole province	827,911	76.3 ± 9.75	21.75 ± 6.29	5.64	19.56	29.51	38.08	41.73
The Pearl-River Delta Region	579,032	75.54 ± 11.02	22.28 ± 6.14	6.10	20.48	30.49	38.58	42.24
The Non-Pearl-River Delta Region	248,879	76.59 ± 9.38	21.55 ± 6.35	5.53	19.17	29.23	37.96	41.65
